# Multiple, giant lymphangectatic pilomatricomas: A rare clinical entity

**DOI:** 10.1002/ccr3.9443

**Published:** 2024-09-17

**Authors:** Fatemeh Mohaghegh, Mohammad Shoushtarizadeh, Elham Tavousi Tabatabaei, Maryam Derakhshan, Ali Ghadirian

**Affiliations:** ^1^ Department of Dermatology, Isfahan University of Medical Sciences Skin Diseases and Leishmaniasis Research Center Isfahan Iran; ^2^ Department of Dermatology University of Pittsburgh School of Medicine Pittsburgh Pennsylvania USA; ^3^ Department of Pathology Isfahan Iran

**Keywords:** benign cutaneous tumors, giant pilomatricoma, lymphangectatic pilomatricomas, pilomatricoma

## Abstract

This case highlights the rare and atypical presentation of giant, multiple pilomatricomas (PMs) with a pseudo‐bullous appearance and lymphangiomatous pathology. It underscores the importance of considering PM in the differential diagnosis of atypical lesions, especially those mimicking cystic or hemorrhagic malignant conditions, to prevent misdiagnosis and ensure appropriate management.

## 1 INTRODUCTION

Pilomatricoma (PM), also known as pilomatrixoma, is a benign tumor originating from the hair follicle matrix.^1^ PM is characterized by the presence of subcutaneous nodules, typically up to 3 cm in diameter, and is most commonly located on the head, neck, and upper extremities.^2^ While these nodules rarely exceed 3 cm,^3^ they can present at any age, with a higher prevalence in children and adolescents, as well as adults over 60 years of age.^4^ Histopathological examination of excisional biopsy specimens reveals basaloid cells transitioning to eosinophilic shadow cells, which are anucleate remnants of epithelial cells.^5^ The prognosis for these lesions is generally favorable.

In this report, we describe an unusual case of giant, multiple PMs with a pseudo‐bullous appearance on the right and left arms and upper back, which histologically displayed a lymphangiomatous patter.

## 2 CASE PRESENTATION

**FIGURE 1:**
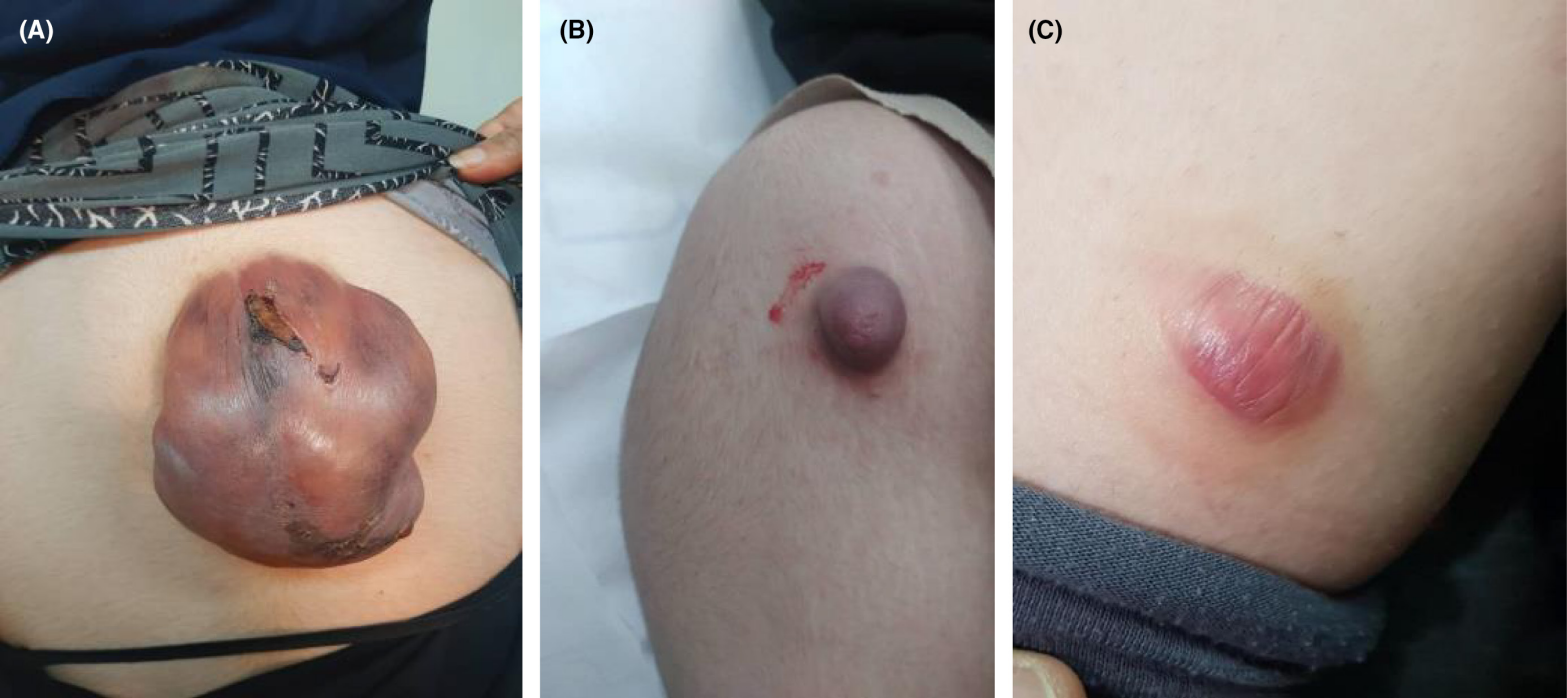
The lesions on the upper back (A), right arm (B) and left arm (C).

**FIGURE 2:**
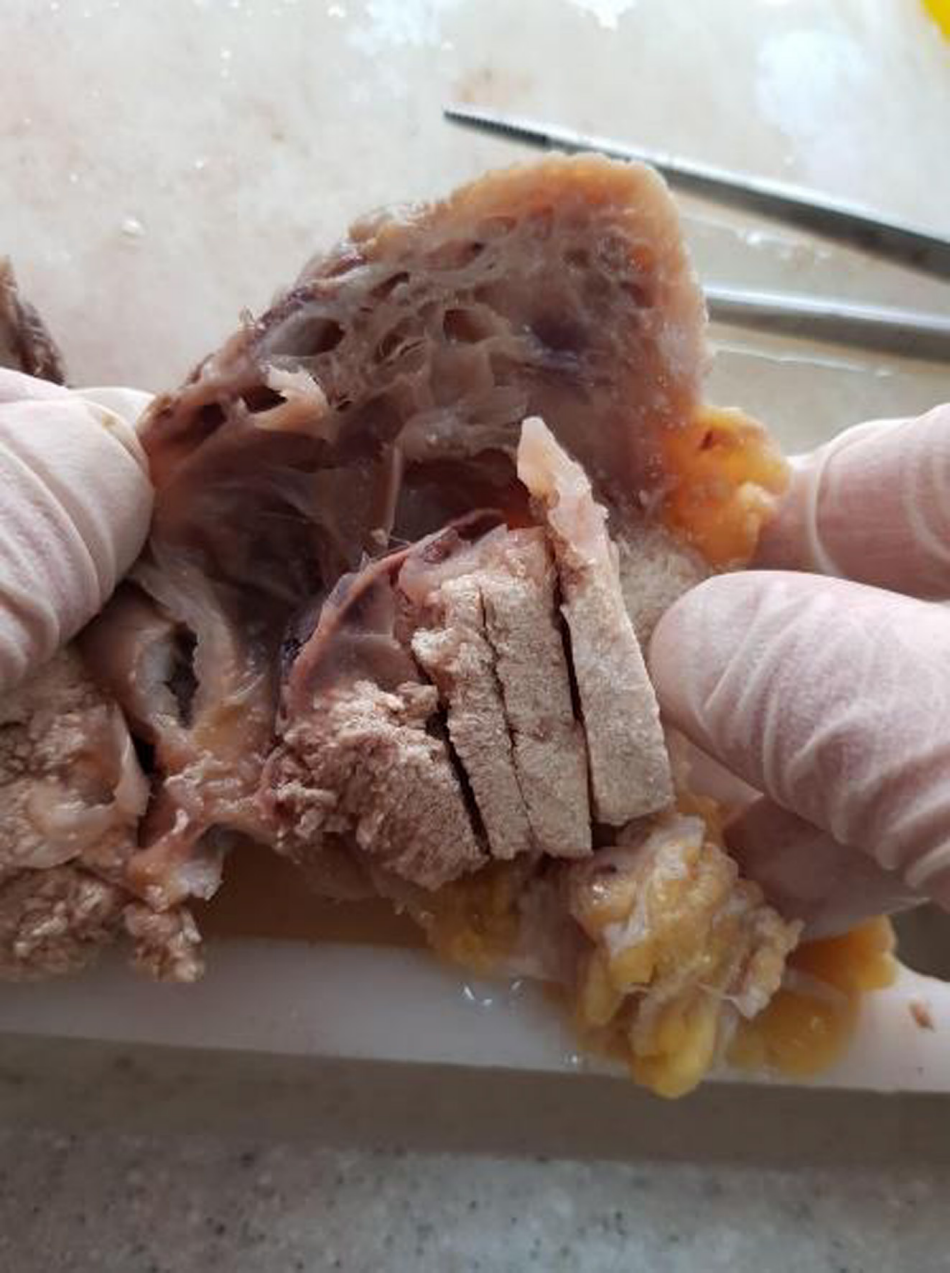
Macroscopic description: Solid cut surface well defined mass overlying by dilated spaces and skin tissue.

**FIGURE 3:**
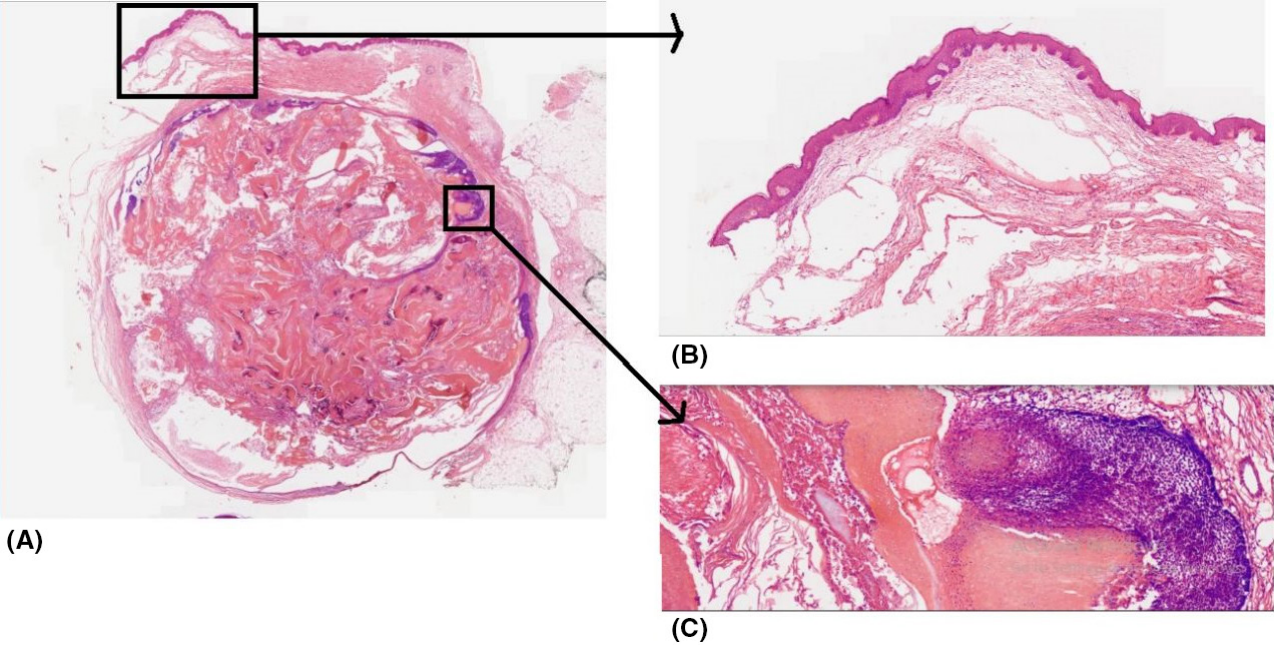
(A) Original magnification: 40, Well circumscribed lesion in the dermis with dilated lymphatic veins overlying the tumor resulting bullous appearance. (B) Original magnification: 100, dilated lymphatic veins. (C) Islands of basaloid cells with eosinophilic keratinized ghost cells.

We present the case of an 18‐year‐old woman with no prior medical history who was referred to the dermatology clinic due to tumors on her right and left arm and upper back. The lesions had been present for approximately 2 years and had grown over the last three to 4 months. The patient reported no pain or trauma prior to the appearance of the swelling. Upon physical examination, a tumoral lesion measuring approximately 15.5 × 15.2 cm was observed on the upper back (Figure 1A–C), a 2.5 × 3.5 cm lesion on the right arm, and a 5 × 5.3 cm lesion on the left arm. In the physical examination, all lesions exhibited a pseudo bullous appearance and were painful upon palpation. They were noted to have a firm consistency and a red‐brown tint, without signs of ulceration or fixation. The lesion on the upper back had progressively increased in size over the last month, due to hemorrhage, from 15.5 × 15.2 cm to 18.4 × 17.5 cm and had transformed into a hemorrhagic cystic lesion. An MRI report, which excluded any vascular abnormalities, was followed by an excisional biopsy of the lesions. The specimens were sent for microscopic examination with a differential diagnosis that included vascular lesions, neurofibroma, adnexal tumor, and cystic lesions. Grossly, the largest tumor in the upper back presented as well‐defined masses with a solid cut surface covered by skin tissue, and some dilated spaces were apparent between the skin and main lesion (Figure 2). Microscopic examination of all lesions revealed tissue with solid nests of basaloid cells underlying abrupt trichilemmal‐type keratinization with ghost cells and marked dilation of superficial lymphatics overlying the tumor. A loss of elastic fibers was also noted. The diagnosis for all mass was bullous (lymphangectatic) PM (Figure 3A–C). Since the treatment was surgical excision, postoperative follow‐up has shown no relapse or development of a new lesions to date.

## 3 DIFFERENTIAL DIAGNOSIS, INVESTIGATIONS, AND TREATMENT

PM is a relatively rare benign skin tumor. Up to 40% of PMs manifest before 10 years of age, with more than 60% of cases presenting in the first two decades.^6^ There is a higher incidence in females.^7^ While PMs typically occur in the head and neck region, this report documents lesions in the arm and upper back. Classically, a PM presents as a single, rigid to firm, painless dermal or subcutaneous nodule, ranging from 0.5 to 3 cm in size. Lesions exceeding 10 cm, termed “giant PM,” are exceedingly rare.^8^ The literature contains only a handful of cases involving giant PMs greater than 10 cm in diameter.^9^ The occurrence of multiple PMs is also rare and may be associated with myotonic dystrophy, Gardner syndrome, Turner syndrome, trisomy 9, spina bifida, or sarcoidosis.^10^

The differential diagnosis for such lesions may include squamous cell carcinoma (SCC), sarcoma, spinocellular carcinoma, lymphoma, dermatofibrosarcoma, dermoid cysts, branchial cleft remnants, pre‐auricular sinuses, sebaceous cysts, hemangiomas, or malignant soft tissue tumors.^2^ MRI findings of PM typically present as a well‐defined soft tissue mass involving the cutaneous and subcutaneous layers, displaying iso/hypointense T1 signals relative to muscle, heterogeneous T2 signals, and heterogeneous enhancement. Our case is distinguished by the number of lesions, their diverse appearances, some atypical localizations, and the unconventional presentation of PM. For complete removal of typical lesions, a narrow excisional margin is generally sufficient, whereas for giant PMs, a wider excision margin of 1–2 cm, guided by preoperative diagnostic imaging, is advised.^11^ The incidence of PM with a bullous appearance is estimated to be between 3% and 6%.^12^ While PM may be linked to genetic disorders such as myotonic dystrophy and Turner syndrome, pseudobullous PM does not appear to be associated with genetic anomalies, as reviewed by Chen et al.^13^ Histologically, pseudobullous PM typically exhibits nests of basophilic and shadow cells (ghost cells), copious dilated lymphatic vessels, and lymphoedema in the superficial dermis. The conventional differential diagnosis includes lymphangioma, malignant tumors, and bullous morphea.^12^, ^13^ In cases of bullous PM, most exhibit dilated lymphatic vessels and severe lymphedema in the dermis overlying a typical PM.^14^ Currently, the pathogenesis of pseudobullous PM remains elusive. Some hypotheses propose that the elastinolytic enzymes secreted by tumor cells, along with lytic products, may contribute to the degradation of elastic fibers and the subsequent dilation and destruction of lymphatic vessels, culminating in lymph fluid accumulation within the dermis, thus creating a bullous presentation.^15^

## 4 DISSCUSION

The pathogenesis of bullous PM remains poorly understood. The prevailing hypothesis suggests that PM leads to obstruction of lymphatic vessels and consequent lymphatic fluid congestion. This condition is believed to cause dilation of the lymphatic vessels, lymph leakage, and resultant dermal edema. It is postulated that external pressure may contribute to the development of lymphedema. Consequently, bullous PM is also referred to as lymphangiectatic PM.^4^ Giant PM represents a distinct clinical variant of PM, characterized by its substantial size, common location in the parotid area, and frequent ulceration, which may mimic malignant or parotid tumors clinically. Recognizing and differentiating this entity from pilomatrical carcinoma is crucial.^16^ To date, literature comprises 53 articles documenting 70 cases of giant PM, indicating a higher prevalence in adult males. These lesions typically present as a solitary nodule, persisting anywhere from 1 month to 40 years, and can vary in size, with the largest reported lesion measuring 34 cm in diameter.^17^ Nearly 40% of reported giant PMs exhibit ulceration, and their predilection for the parotid region often leads to an initial misdiagnosis of malignant^18^ or parotid neoplasms,^19^ including entities such as cutaneous squamous cell carcinoma, dermatofibrosarcoma protuberans, cutaneous metastases, pleomorphic adenoma, and malignant parotid tumors.^18^ Due to diagnostic challenges, more than half of the reported cases required imaging studies or cytological evaluation through fine needle aspiration (FNA). The imaging modalities frequently requested include computed tomography and MRI, typically revealing a well‐defined soft tissue mass with heterogeneous calcifications, abundant tortuous vessels, but no invasion of adjacent structures. Key cytological features indicative of PM on FNA include basaloid and ghost cells, with multinucleated giant cells, nucleated squamous cells, and calcium deposits also providing diagnostic clues.^20^ The occurrence of pseudo bullous PM is acknowledged in select literature, as noted in our case report.^21^, ^22^

Our case is distinguished by the presence of multiple lesions and one giant lesion, displaying a pseudo‐bullous appearance and lymphangiomatous pathology not previously described in the literature. The clinical presentation of our lesion resembled cystic, hemorrhagic, and bullous malignant lesions, marking it as clinically atypical. Thus, PM should be considered in the differential diagnosis of lesions exhibiting cystic and pseudo bullous features.

In summary, the development of multiple PMs in our otherwise healthy patient may be attributed to a combination of genetic, environmental, immunological, and possibly hormonal factors. The lymphangiomatous pattern observed in the histopathological examination raises the possibility of localized lymphatic obstruction contributing to the formation of these atypical lesions. Given that PMs are typically benign and solitary, the presentation of multiple, giant lesions in atypical locations as seen in this case suggests a potential deviation from the conventional pathogenesis. One hypothesis is that subtle, undiagnosed genetic mutations or epigenetic changes may predispose certain individuals to an increased risk of developing multiple PMs. Additionally, environmental factors, such as chronic low‐level exposure to irritants or minor, unnoticed trauma, may have acted as triggers for tumor formation. The role of the immune system in this process cannot be discounted, as local inflammatory responses, even in the absence of systemic symptoms, might contribute to the proliferation of PMs. Furthermore, the patient's age and hormonal status could have played a role, particularly if there were fluctuations or imbalances that contributed to the tumor development during critical periods of growth.

Finally, the lymphangiomatous features observed in this case suggest that lymphatic obstruction, whether primary or secondary to other factors, may play a crucial role in the pathogenesis of giant PMs with a pseudo‐bullous appearance. The dilation of lymphatic vessels and subsequent accumulation of lymph fluid may create a microenvironment that facilitates tumor growth and alters its clinical presentation. While the exact etiology remains speculative, this case highlights the importance of considering a multifactorial origin in the development of multiple PMs and emphasizes the need for further research to elucidate these complex mechanisms.

## AUTHOR CONTRIBUTIONS

**Fatemeh Mohaghegh:** Conceptualization; data curation; formal analysis; funding acquisition; methodology; project administration; supervision; validation; visualization; writing – review and editing. **Mohammad Shoushtarizadeh:** Conceptualization; formal analysis; methodology; resources; visualization; writing – original draft; writing – review and editing. **Elham Tavousi Tabatabaei:** Writing – review and editing. **Maryam Derakhshan:** Data curation; formal analysis; writing – review and editing. **Ali Ghadirian:** Data curation; writing – original draft.

## FUNDING INFORMATION

The authors received no financial support for this study.

## CONFLICT OF INTEREST STATEMENT

The authors declare no conflicts of interest.

## ETHICS STATEMENT

This study has obtained ethical approval from the Isfahan University of Medical Sciences.

## CONSENT

Written informed consent was obtained from the patient to publish this report in accordance with the journal's patient consent policy.

## Data Availability

All data used and analyzed during this study are available from the corresponding author upon reasonable request.
